# Selective whole genome amplification of *Plasmodium malariae* DNA from clinical samples reveals insights into population structure

**DOI:** 10.1038/s41598-020-67568-4

**Published:** 2020-07-02

**Authors:** Amy Ibrahim, Ernest Diez Benavente, Debbie Nolder, Stephane Proux, Matthew Higgins, Julian Muwanguzi, Paula Josefina Gomez Gonzalez, Hans-Peter Fuehrer, Cally Roper, Francois Nosten, Colin Sutherland, Taane G. Clark, Susana Campino

**Affiliations:** 1 Faculty of Infectious and Tropical Diseases, London School of Hygiene and Tropical Medicine, London, UK; 2 Public Health England (PHE) Malaria Reference Laboratory, London School of Hygiene and Tropical Medicine, London, UK; 3 Shoklo Malaria Research Unit, Mahidol-Oxford Tropical Medicine Research Unit, Faculty of Tropical Medicine, Mahidol University, Mae Sot, Thailand; 4 Department of Pathobiology, Institute of Parasitology, University of Veterinary Medicine, Vienna, Austria; 5 Centre for Tropical Medicine and Global Health, Nuffield Department of Medicine, University of Oxford, Oxford, UK; 6 Faculty of Epidemiology and Population Health, London School of Hygiene and Tropical Medicine, London, UK; 7 Department of Infection Biology, London School of Hygiene and Tropical Medicine, London, UK

**Keywords:** Genetics, Population genetics, Sequencing, Parasitology, Parasite biology, Parasite genomics

## Abstract

The genomic diversity of *Plasmodium malariae* malaria parasites is understudied, partly because infected individuals tend to present with low parasite densities, leading to difficulties in obtaining sufficient parasite DNA for genome analysis. Selective whole genome amplification (SWGA) increases the relative levels of pathogen DNA in a clinical sample, but has not been adapted for *P. malariae* parasites. Here we design customized SWGA primers which successfully amplify *P. malariae* DNA extracted directly from unprocessed clinical blood samples obtained from patients with *P. malariae*-mono-infections from six countries, and further test the efficacy of SWGA on mixed infections with other *Plasmodium* spp. SWGA enables the successful whole genome sequencing of samples with low parasite density (i.e. one sample with a parasitaemia of 0.0064% resulted in 44% of the genome covered by ≥ 5 reads), leading to an average 14-fold increase in genome coverage when compared to unamplified samples. We identify a total of 868,476 genome-wide SNPs, of which 194,709 are unique across 18 high-quality isolates. After exclusion of the hypervariable subtelomeric regions, a high-quality core subset of 29,899 unique SNPs is defined. Population genetic analysis suggests that *P. malariae* parasites display clear geographical separation by continent. Further, SWGA successfully amplifies genetic regions of interest such as orthologs of *P. falciparum* drug resistance-associated loci (*Pfdhfr, Pfdhps, Pfcrt, Pfk13* and *Pfmdr1*), and several non-synonymous SNPs were detected in these genes*.* In conclusion, we have established a robust SWGA approach that can assist whole genome sequencing of *P. malariae,* and thereby facilitate the implementation of much-needed large-scale multi-population genomic studies of this neglected malaria parasite. As demonstrated in other Plasmodia, such genetic diversity studies can provide insights into the biology underlying the disease and inform malaria surveillance and control measures.

## Introduction

Malaria, a mosquito-borne disease caused by *Plasmodium* parasites, is a continuing threat to global health. There were an estimated 228 million cases and 405,000 deaths in 2018^1^. The majority of mortality events are due to *P. falciparum* malaria and therefore disease control and elimination efforts have primarily targeted this species. Molecular surveillance has demonstrated that non-falciparum malaria has been underestimated by microscopy diagnosis^2–5^, and rapid diagnostic tests (RDT), which are unable to diagnose non-falciparum malaria to the species level^6,7^. Molecular studies are beginning to demonstrate alarmingly high levels (4–24%) of *P. malariae* mono- and co-infections across continents^2,8–12^.

*P. malariae* infections commonly present with mild or no symptoms, however, severe disease, including anaemia, renal pathologies, and splenomegaly^13–17^ can occur, complications which can prove fatal^16^. *P. malariae* infections present with quartan fevers with parasites that can remain in the host for decades^13,18,19^ . This persistence is a threat to disease elimination strategies^19^. Severe *P. malariae* infections are commonly treated with an Artemisinin Combination Therapy (ACT), similar to *P. falciparum* infections in the same region^18^. The high prevalence of mixed infections with *P. falciparum* and *P. vivax* means that populations of *P. malariae* may have been experiencing substantial drug pressure. Several reports have described *P. malariae* parasites that have not been cleared after treatment with standard antimalarials^8,17,20^ or have initiated successful infections despite effective chemoprophylaxis^21^, leading to fears of reduced drug efficacy.

Advances in whole genome sequencing (WGS) technologies now allow for large scale genome diversity studies. Such studies in *P. falciparum* and *P. vivax* have provided significant new understanding of the structure of parasite populations, intra- and inter-population genomic diversity, and identified genomic regions under selective pressure, such as drug resistance associated genes^22–25^. However, to date only a few complete genomes have been assembled for *P. malariae* (n = 5; genome size 31.9 Mb)^26,27^, which have led to insights into genome structure including species-specific gene expansions, causing the characteristically large genome of *P. malariae*^27^. One expansion of note is a family encoding transmembrane domain proteins, known as *Pm-fam,* containing *fam-m* and *fam-l* genes, which are hypothesised to be involved in host–pathogen interactions and are unique to *P. malariae* parasites^26,27^. To date, investigations of *P. malariae* genetic diversity have used microsatellite data and demonstrated considerable levels of genetic diversity and differences between and within populations from different countries^28,29^. However, microsatellite markers reflect only a minority of the genome (< 0.1%), and further investigation using WGS data is needed to explore genetic diversity and population structure across endemic regions.

A major challenge in performing WGS studies using clinical parasite isolates is the difficulty in obtaining sufficient *Plasmodium* DNA from infected individuals. This is due to low parasite densities and the presence of human DNA from host lymphocytes and other circulating nucleated cells. For *P. malariae,* genome studies are further complicated by the lack of an in vitro culture method for this parasite species. Until now, WGS data for *Plasmodium* parasites has been obtained using DNA extracted from venous blood of clinical cases that were pre-filtered to remove human leukocytes, in order to reduce the amount of co-extracted human DNA^30^. This methodology is efficient when parasite densities are high, however, this is not the case for the majority of *P. malariae* infections, particularly asymptomatic individuals, where this approach would not yield sufficient parasite DNA for WGS. Recently, a selective whole genome amplification (SWGA) strategy has been used to successfully sequence *P. falciparum*, *P. vivax* and *P. knowlesi* genomes from non-filtered blood and from dried blood spots of clinical samples^31–33^. The SWGA method uses oligonucleotide primers that preferentially bind with high frequency to the pathogen DNA, and rarely bind to the host genome^34^. The high fidelity Phi29 polymerase, which works through multiple displacement amplification (MDA), is used to amplify large segments (~ 70 kb) of DNA, primed by the SWGA oligonucleotides.

The unique but poorly understood characteristics of the *P. malariae* parasite, and the threat of unpredictable drug resistance, indicate a need for better understanding of the biological features of this neglected species. Knowledge of the complexity and variability of the *P. malariae* genome, and comparative studies with the well characterised *P. falciparum* and *P. vivax* genomes^23–25^, could provide insights into the biology of this human parasite species. Here, we adapt and validate the SWGA approach for amplification of the *P. malariae* genome, successfully processing and sequencing 19 clinical samples. After selecting 18 high quality samples, we demonstrate that the resulting WGS data can be used to assess genetic diversity in *P. malariae* genes orthologous to known drug resistance markers in other species, and to inform population structure. In doing so, we provide proof-of-principle for large-scale WGS studies using blood samples collected from malaria endemic regions to inform malaria control efforts, and provide new molecular information for development of diagnostics, vaccines and drugs.

## Results

### SWGA enriches *P. malariae* DNA and increases WGS data coverage

We performed SWGA using a designed primer set (denoted as Pmset1) consisting of five primers (see S1 Table) that preferentially bind the *P. malariae* genome (average binding sites located once every 2.9 kb within the *P. malariae* genome, compared to once every 45.1 kb in the human genome). For successful selective amplification it is essential that the binding sites are in close proximity in the parasite genome and spaced further apart in the human genome^35^. Using two test samples (PM_THA_001 and PM_THA_002), we demonstrate that Pmset1 successfully amplifies the *P. malariae* genome, allowing for higher quality WGS data in comparison to non-amplification (S1 Fig.). Whilst all four samples were sequenced at a similar depth, we observed that amplified samples have a significant increase in coverage, with a mean 18.6-fold increase in the percentage of the genome covered with ≥ 5 reads when compared to non-amplification (S2 Table). The increase in genome coverage seen with SWGA allows for greater detection of SNPs which can be used for downstream population genetics analysis. As a result, there was an 800- to 13,000-fold increase in the number of callable SNPs detected in samples amplified using Pmset1 (S2 Table).

**Figure 1:**
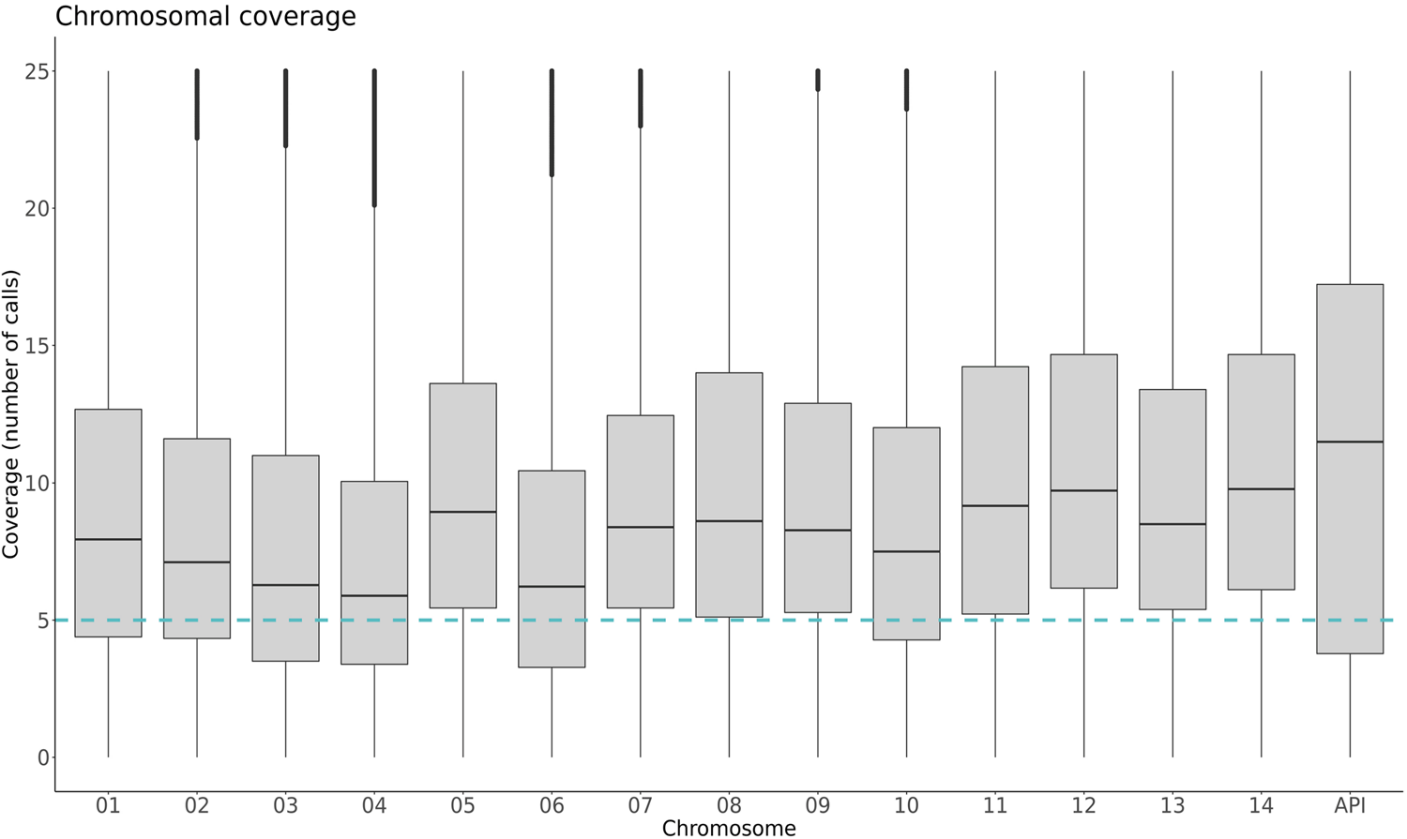
Sequencing coverage by chromosome after SWGA. The distribution of coverage for each position within the genome between 18 high quality samples, among the 14 nuclear chromosomes and the organellar apicoplast (the mitochondrial coverage plot is excluded due to high coverage). The blue horizontal line represents the recommended coverage cut-off point for SNP calling (≥ 5 reads).

After validation of Pmset1, 17 additional clinical samples were amplified using Pmset1 and underwent WGS. One sample (PM_THA_009), with a low parasitaemia of 0.0016% presented with low coverage after the first sequencing run (27% genome covered ≥ 5 reads), this sample was re-sequenced, and the second run had better results (44% genome covered ≥ 5 reads) (S2 Table). The two sequencing runs were combined to generate PM_THA_009com (52% genome covered ≥ 5 reads). Excluding the separate runs for PM_THA_009, and one sample with low genome coverage (PM_LBR_003), the remaining samples had an average of 67.4% (± 15%) of the genome covered by ≥ 5 reads (S2 Table). The coverage profile after amplification was uneven, as reported for other Plasmodia^32^, but generally, across all chromosomes, reaching coverage above the recommended cut off point for SNP calling (five reads or above) (Fig. 1). Coverage of the mitochondria was variable but consistently high in comparison to other chromosomes (mean: 26-fold coverage). The average chromosomal coverage of the two unamplified samples was much lower, with only 0.82% of the genome with a coverage ≥ 5 reads (S2 Fig).

### SWGA is dependent on the initial parasitaemia of a sample

**Figure 2:**
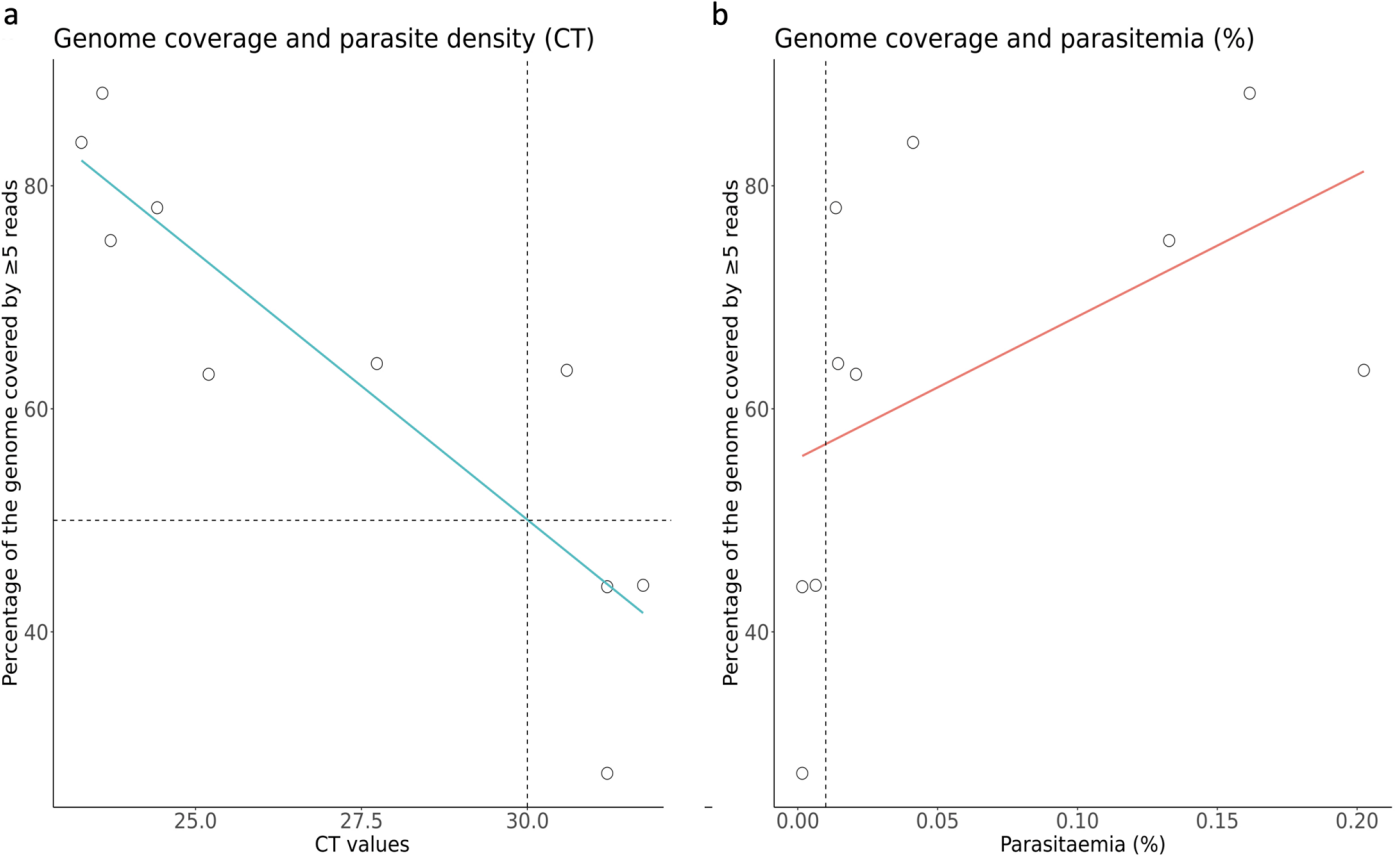
Correlation between parasite density and genome coverage. (**a**) Nine samples (amplified with SWGA approach) were used to assess the effect of parasite density (as measured by CT values obtained from qPCR) on the proportion of the genome covered by five or more reads. Each point demonstrates a single sample, with a lowess line of best fit. The dashed horizontal line represents a cut off of 50% of the genome covered by 5 or above, and the dashed vertical line indicates the suggested CT cut-off of 30. (**b**) The same plot is shown using parasitaemia as the measure of parasite density (percentage of RBCs parasitized). Parasitaemias range from 0.0004% to 0.2024%, and the vertical dashed line represents the suggested parasitaemia cut-off of 0.01%.

To determine a potential limit of parasitaemia for WGS, a measure of genomic coverage was assessed in nine Thailand samples for which parasitaemia data was available (range of parasitaemia: 0.0004% to 0.2024%). We determine a parasitaemia limit using both microscopy estimates and cycle threshold (CT) values calculated using the qPCR method^36^. We plotted the CT values of each sample alongside the percentage of the genome that was covered by ≥ 5 reads. We determined that a CT value of 30 will lead to an estimate of 50% of the genome covered by ≥ 5 reads (Fig. 2a). Coverage results are unpredictable below this limit, however, as with PM_THA_001, sequence data may be usable below this limit. When using percentage parasitaemia, we verified that all sequence data from parasite densities higher than 0.01% (400 parasites/ul) led to > 50% of the genome covered by five or more reads; this is a lower limit than previously defined for *P. falciparum,* and *P. knowlesi*^32,37^ (Fig. 2b). For difficult samples with lower parasitaemia it is possible to improve genome coverage by performing independent SWGA reactions and by increasing sequence data, as observed previously for *P. vivax*^31^, and also demonstrated here for PM_THA_009, for which merging data lead to > 50% of genome covered with at least 5 reads (S2 Table).

### Determining and excluding hypervariable regions

**Figure 3:**
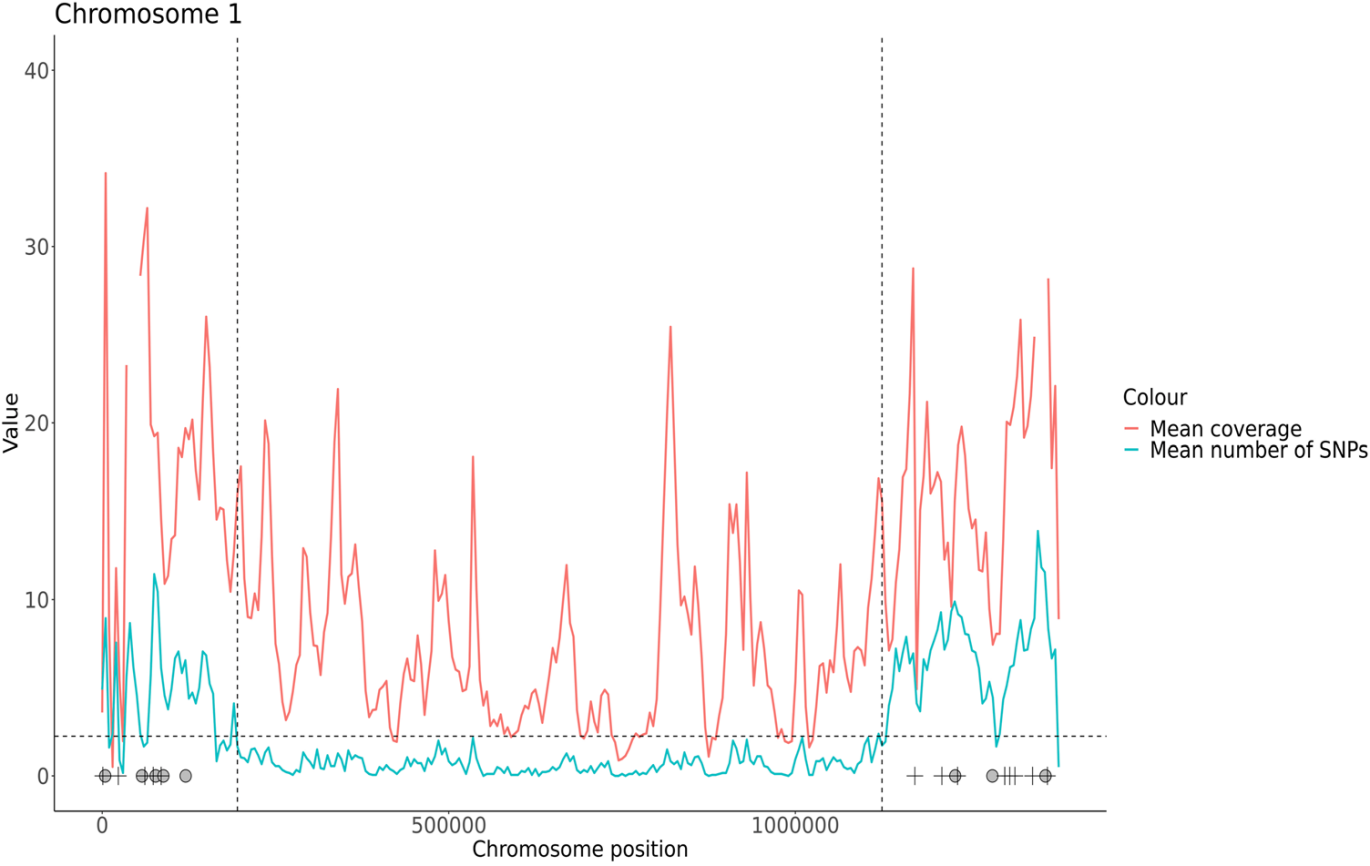
Defining and excluding subtelomeric regions, an example with chromosome 1. The average coverage (pink) and number of SNPs (blue) between all 18 samples for chromosome 1. The black dashed horizontal line demonstrates the previously chosen SNP limit per 5 kb window (as defined in S2 Fig.). Black dashed lines are placed at the suggested hypervariable region cut-off points, where clusters of windows demonstrating > 2.25 SNPs are seen. The midpoints of *Pm-fam* gene families are annotated; *Pm-fam-l* gene positions are denoted by a black plus, whilst *Pm-fam-m* gene positions are denoted with a grey circle. (S3 Fig. for all chromosomes, S3 Table for coordinates).

Many *Plasmodium* species are known to contain large regions of repetitive sequences within the subtelomeres, which is exaggerated in the case of *P. malariae,* leading to an enlarged genome in comparison to other species^27^. We defined the core genome by both excluding regions with > 2.25 SNPs on average per 5 kb window (S3 Fig.) or containing *Pm-fam* genes (Fig. 3, S4 Fig., core genome coordinates are listed in S3 Table), to leave a total core genome size of 23,960,057 bases (81% of the total PmUG01 reference genome).

### Genetic diversity and population structure

We investigated the multiplicity of infection (MOI) in all samples using the core genome, initially through determining the proportion of SNPs that were heterozygous, alongside running *estMOI*^38^ for each sample which calculates the percentage of the genome that supports a MOI of 1 (S2 Table, S5 Fig.). The samples were *P. malariae* mono-infections, that is, where no other Plasmodium species were detected by qPCR. However, it is possible that > 1 clone of *P malariae* is present in a sample i.e. polyclonal. Using this sample set, three isolates displayed evidence of polyclonal infections (PM_LBR_002, PM_UGA_007 and PM_THA_012). This observation was confirmed by assessing the minor allele frequency (MAF) distribution of these isolates, where they presented with a higher proportion of SNPs with a non-reference MAF in the range 0.2 to 0.8 (S6 Fig.). For these three isolates only the major allele strain in each isolate was used in further population genetics analysis.

A total of 868,476 genome-wide SNPs were found within the 18 high quality samples (average of 48,249 SNPs per sample), of which 194,709 were unique. However, as with other *Plasmodium* spp., the subtelomeric region of the *P. malariae* genome contains large sections of repetitive DNA sequence^27^. These regions are problematic when interpreting WGS data from short-read technologies such as Illumina as short reads are likely to be aligned to incorrect regions along the reference genome, leading to deceptively high coverage and number of SNPs.

After removing hypervariable regions, we analysed the core genome (see S3 Table for coordinates) of 18 samples (≥ 40% of the genome covered by ≥ 5 reads) and identified 29,899 unique SNPs (mean: 5,810 ± 2,229 SNPs per sample) for downstream population genetic analysis. We found that geographically proximal samples displayed less pairwise diversity than geographically separated samples, with parasites from Thailand appearing more closely related to each other than to parasites obtained from Africa. Nucleotide diversities (π) > 3 × 10^−4^ nucleotide differences per site are only seen when comparing samples between Thailand and Africa, and π < 2 × 10^−4^ was only seen when comparing samples within Thailand or Africa (S4 Table).

**Figure 4:**
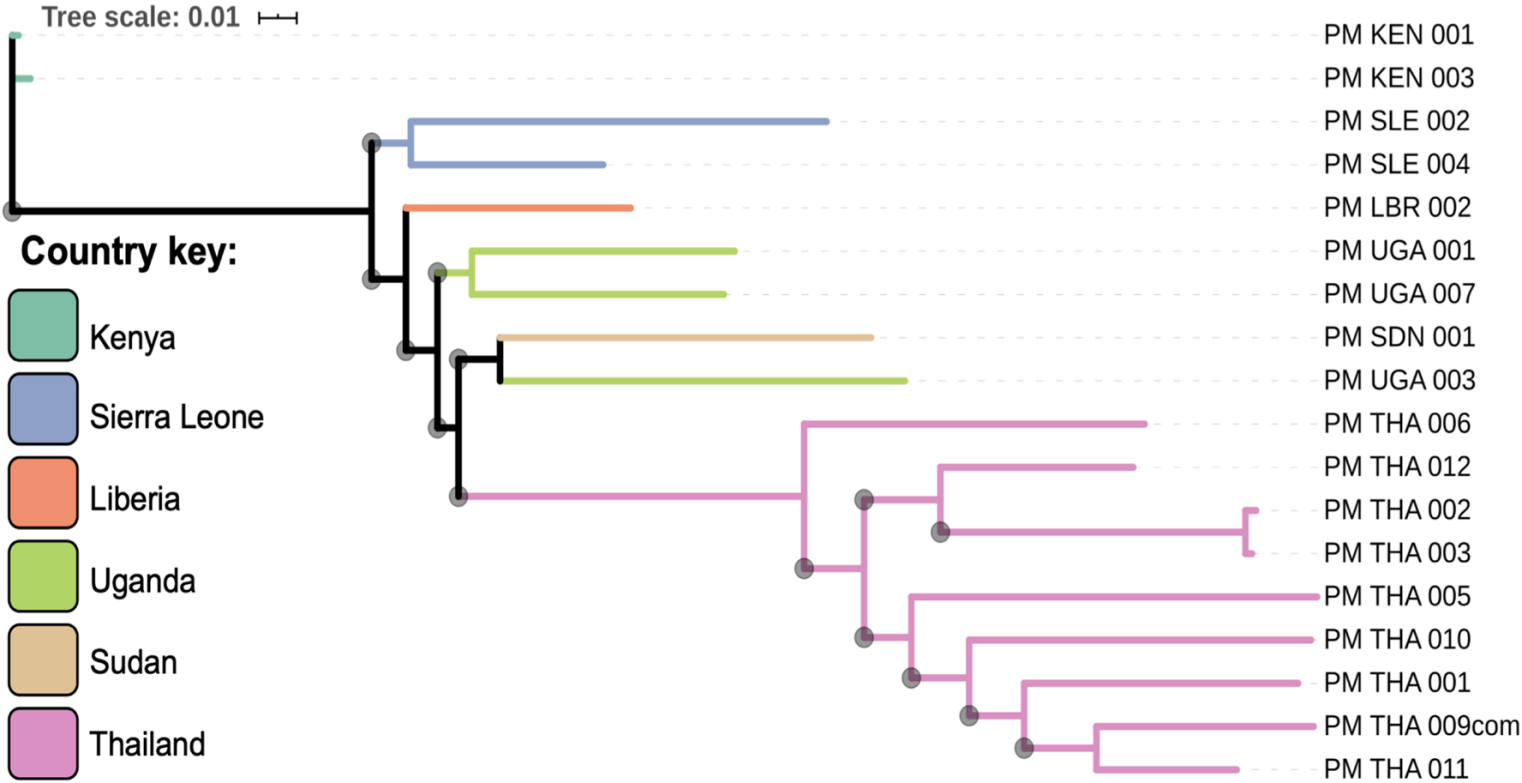
Population structure of *P. malariae* parasites. A maximum likelihood (ML) tree was generated using 29,899 unique SNPs from 18 amplified and sequenced samples (> 40% genome with at least fivefold coverage). The ML tree is unrooted and was generated using *Iqtree*^39^ with *Modelfinder* software used to select the best model of substitution^40^. Horizontal branch lengths are drawn to scale demonstrating the number of substitutions per position, and branch bootstrap values (determined using *UFBoot2*^41^) above 50 are denoted with a grey circle at the start of each branch. The tree was visualised in *iTOL*^42^, and branches were coloured by country (country codes: KEN = Kenya, SLE = Sierra Leone, LBR = Liberia, UGA = Uganda, THA = Thailand, SDN = Sudan).

A maximum-likelihood tree was constructed using core genome SNP data and demonstrates clear regional separation of *P. malariae* parasites, with samples from the African continent clustering together, and independently from samples originating in Thailand (Fig. 4).

### Genetic variation in in orthologs of known *P. falciparum* genes associated with drug resistance

**Figure 5:**
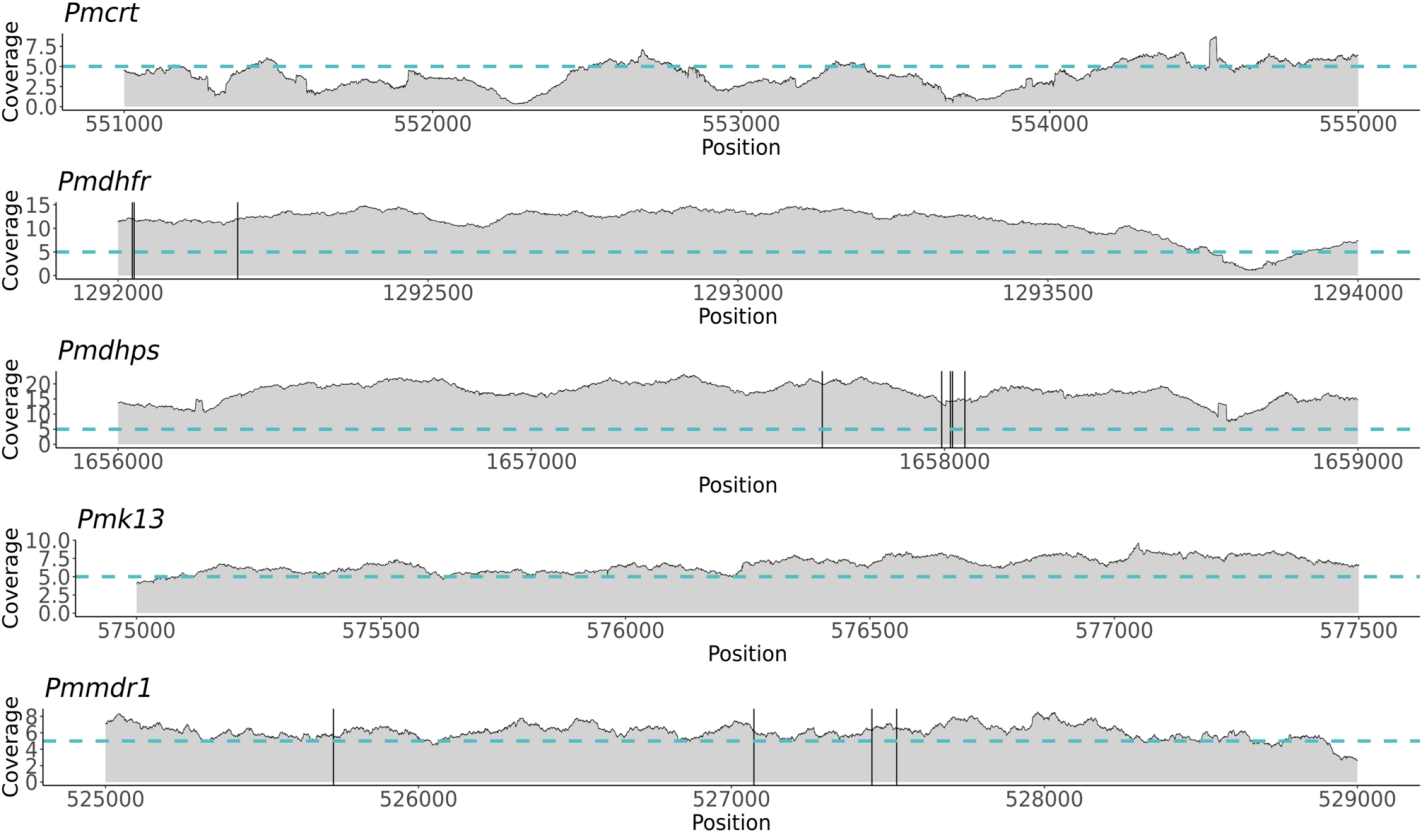
Average sequencing coverage and SNPs found within *P. malariae* orthologues of *P. falciparum* drug resistance associated genes. Average sequencing coverage for 18 samples across 5 genes is represented. The blue horizontal line indicates the coverage of 5 reads and black vertical lines are locations of SNPs (*Pmdhfr:* 1,292,023, 1,292,026 and 1,292,193*; Pmdhps:* 1,657,704, 1,657,993, 1,658,014, 1,658,019 and 1,658,049; *Pmmdr1:* 525,728, 527,072, 527,449 and 527,528).

*P. malariae* parasites are commonly subject to antimalarial treatments, therefore we investigated the coverage and prevalence of mutations in orthologs of known *P. falciparum* genes associated with drug resistance (*Pfcrt, Pfdhfr, Pfdhps, Pfk13* and *Pfmdr1;* gene IDs are in S5 Table). SNPs were only found in *Pmdhfr* (n = 3; 2 non-synonymous), *Pmdhps* (n = 5; 1 non-synonymous) and *Pmmdr1* (n = 4, 2 non-synonymous) (Fig. 5, Table 1). SNPs within *Pmdhfr* at positions 1,292,026 and 1,292,193 in chromosome 5 appear to be more common globally than other SNPs, whereas SNPs within *Pmdhps* and *Pmmdr1* appear to be more prevalent in Thailand than Africa (Table 2). All of the non-synonymous mutations found within *Pmdhfr* led to amino acid alterations (F57L, R58S and N114S) at positions that align with known drug-resistance associated positions within the *Pfdhfr* ortholog (C59R and S108N respectively) upon amino acid allignment (Table 1, S7 Fig.)^43^. In addition, the mutation at position 527,528 within *Pmmdr1* (chromosome 10), which leads to the amino acid substitution L1063F, aligns in close proximity to N1042D in the *Pfmdr1* ortholog that is associated with quinine resistance, and increased mefloquine and artemisinin susceptibility (Table 1, S7 Fig.)^44^.

**Table 1:** Single nucleotide polymorphisms (SNPs) detected in *Pmdhfr*, *Pmdhps* and *Pmmdr1* genes, and their downstream effects.

Gene	Position	Ref	SNP1	Effect	Change in codon	Change in amino acid	Proportion of SNP1	SNP 2	Effect	Change in codon	Change in amino acid	Proportion of SNP2
*Pmdhfr*	1,292,023*	**C**	**A**	**NS**	**ttC/ttA**	F57L*	**0.17**	**G**	**NS**	**ttC/ttG**	F57L*	**0.11**
	1,292,026*	A	G	S	agA/agG	R58	0.28	**C**	**NS**	**agA/agC**	R58S*	**0.67**
	1,292,193*	**A**	**G**	**NS**	**aAc/aGc**	N114S*	**0.78**	/	/	/	/	
*Pmdhps*	**1,657,704**	**C**	**T**	**NS**	**Gtg/Atg**	**V121M**	**0.06**	/	/	/	/	
	1,657,993	C	T	I	/	/	0.28	/	/	/	/	
	1,658,014	A	G	I	/	/	0.11	/	/	/	/	
	1,658,019	A	T	I	/	/	0.22	C	I	/	/	0.06
	1,658,049	T	A	I	/	/	0.06	/	/	/	/	
*Pmmdr1*	525,728*	**T**	**G**	**NS**	**ttA/ttC**	L1063F*	**0.11**	/	/	/	/	
	527,072	C	T	S	ttG/ttA	L615	0.22	/	/	/	/	
	**527,449**	**G**	**T**	**NS**	**Ctt/Att**	**L490I**	**0.06**	/	/	/	/	
	527,528	G	A	S	agC/agT	S463	0.33	/	/	/	/	

*Amino acid alterations that lie in close proximity to known *P. falciparum* resistance mutations. SNPs leading to non-synonymous (NS) mutations are in bold, whilst intronic (I) or synonymous (S) mutations are unbolded.

**Table 2:** Distribution of SNPs in *Pmdhfr*, *Pmdhps* and *Pmmdr1* among 18 samples from Africa and Thailand.

Gene	*Pmdhfr*			*Pmdhps*					*Pmmdr1*			
Position	1292023*	1292026*	1292193*	1657704	1657993	1658014	1658019	1658049	525728	527072	527449	527528*
Reference allele	**C**	**A**	**A**	**C**	**C**	**A**	**A**	**T**	**T**	**C**	**G**	**G**
PM_THA_001	/	C	G	/	/	/	/	/	G	–	–	–
PM_THA_002	/	C	G	/	T	/	/	/	/	/	/	/
PM_THA_003	/	C	G	/	T	A/G	A/T	/	N	N	N	N
PM_THA_005	/	C	G	/	T	/	A/T	/	G	/	/	/
PM_THA_006	/	C	G	/	T	A/G	A/T	/	/	N	/	N
PM_THA_009com	/	C	G	/	/	/	A/C	A/T	/	T	/	A
PM_THA_010	/	C	/	/	T	/	A/T	/	/	T	T	A
PM_THA_011	/	C	G	/	/	/	/	/	/	N	/	A
PM_THA_012	/	C	/	/	/	/	/	/	/	T	/	A
PM_KEN_001	A	G	G	/	/	/	/	/	N	/	N	N
PM_KEN_003	A	G	G	/	/	/	/	/	N	N	N	–
PM_LBR_002	/	C	G	C/T	/	/	/	/	/	/	/	A/G
PM_SDN_001	G	G	G	/	/	/	/	/	N	–	N	N
PM_SLE_002	/	C	G	/	/	/	/	/	/	T	/	/
PM_SLE_004	/	C	/	/	/	N	N	N	N	N	N	N
PM_UGA_001	N	N	/	/	N	N	N	N	–	–	N	N
PM_UGA_003	A	G	G	/	/	/	/	/	/	/	/	A
PM_UGA_007	G	G	G	/	/	/	/	/	N	N	N	N

/ denote Wild-type alleles (the allele observed in the PmUG01 reference genome).

N denotes no coverage at this position.

*Positions lead to amino acid substitutions that align with drug resistance-associated substitutions observed in *P. falciparum*.

### Mixed infections

*P. malariae* parasites are commonly found in mixed infections with other *Plasmodium* spp.^9,11,12^. This provides a further obstacle for WGS, as not only is the human genome a potential contaminant, but also the other *Plasmodium* species present. We used four further unprocessed clinical blood samples from Thailand which were found to be mixed infections after qPCR^36^ and underwent SWGA to determine whether Pmset1 was specific to only the *P. malariae* genome. Each sample contained varying mixtures of other parasite species present and our results suggest that SWGA is likely to work if *P. malariae* is initially the most prevalent parasite in the mixed infection (i.e. has the lowest CT value) (S6 Table). However, when DNA from other species is present at high concentrations, SWGA may not be effective for amplification of *P. malariae* (S6 Table).

## Discussion

*P. malariae* is a neglected malaria parasite with unique features, such as a longer quartan cycle and the ability to persist in the human host for years or decades^13^. Genetic investigation of this parasite may allow us to understand how *P. malariae* is able to cause chronic infections, why there are accounts of *P. malariae* parasites persisting after treatment with ACT, and why some *P. malariae* infections lead to severe outcomes whilst others remain asymptomatic. Malaria parasite genomics can provide important biological insights to understand this disease, but the difficulty of obtaining sufficient parasite DNA for WGS has been a challenge for genomic studies of *P. malariae*. Here we present the first application of SWGA for this species. We have customized the SWGA approach to successfully amplify *P. malariae* DNA extracted directly from unprocessed blood from clinical samples which were obtained from six different countries. In agreement with others^31,32^, we have demonstrated that the parasitaemia affects the efficiency of SWGA, and recommend using samples with a percentage parasitaemia > 0.01%, which is a lower threshold than reported for other species^31,32,37^. The WGS data generated from SWGA-treated samples is of high quality with good overall coverage, leading to an average of 67.4% (± 15%) of the genome covered by ≥ 5 reads between the 18 samples assessed in this study. Using these samples, we were able to identify 868,476 total SNPs (average 48,249 SNPs per sample), filtered to 104,583 total SNPs after exclusion of hypervariable regions (average of 5,810 SNPs per sample). This is lower than SNP prevalence documented in *P. knowlesi* (115,995 SNPs per sample including hypervariable regions)^37^, yet higher than SNPs found in *P. vivax* (14,463 SNPs per sample before filtering for core genome) after SWGA^31^.

It is important to note that differences in the number of SNPs per sample reported could also be due to differences in the method used for variant calling.

A maximum likelihood tree based on SNP data revealed geographic clusters, with clear separation of African and Asian samples. This geographical clustering is consistent with data for *P. falciparum*^45^ and *P. vivax* parasites^24,45,46^. Similar geographic clustering was observed in the phylogenetic analysis of SNPs in the circumsporozoite gene from *P. malariae* isolates from Africa and Asia^47^. To improve geographical clustering resolution (i.e. by country), the number of samples investigated needs to be increased. Our data suggests that parasites display isolation by distance, therefore country or multi-country regional analysis of *P. malariae* populations could be used in future studies to identify regions under selection in different populations.

We further demonstrate that SWGA successfully amplifies genes orthologous to those associated with drug resistance in *P. falciparum*, and identify SNPs in *Pmdhfr*, *Pmdhps* and *Pmmdr1*. The effects of these SNPs are unknown, and to date, there are no characterised molecular markers of drug resistance in *P. malariae* parasites, even though treatment failures have been reported^19,48^. Despite this*,* potential mutations of interest were found, particularly at positions 1,292,023, 1,292,026 and 1,292,193 in chromosome 5 in the *Pmdhfr* gene. These mutations lead to amino acid substitutions F57L, R58S and N114S respectively, and align almost perfectly with *P. falciparum* amino acid substitutions C59R and S108N which are associated with reduced susceptibility to sulfadoxine/pyrimethamine^49^. The nonsynonymous mutation N114S has been previously reported in two *P. malariae* samples from Thailand and the F57L and R58L mutations have been reported in *P. vivax* samples from several geographical regions^50,51^. In addition, one mutation within *Pmmdr1* at position 525,728 in chromosome 10 leads to amino acid substitution L1063F, which aligns with close proximity to N1042 in the *Pfmdr1* ortholog, associated with reduced susceptibility to quinine and increased susceptibility to mefloquine, halofantrine and artemisinin^44^. It is important to note that whilst treatment failures are seen with *P. malariae* infections, it is not clear whether this is due to mutations within the parasite genome leading to reduced drug efficacy, or perhaps a specific phenotype of this species due to the longer parasite life cycle which may reduce drug absorption^48^; therefore further functional studies are required to determine the effect, if any, of these substitutions.

The subtelomeres, containing the *fam-l* and *fam-m* gene families are of great interest when studying *P. malariae*, as they are unique to this species and are thought to be involved in host-parasite interactions^26^. Unfortunately, sequence analysis of these regions is notoriously difficult using short-read technologies, therefore longer-read sequencing will be needed to further investigate these regions.

In conclusion, the SWGA approach offers a fast, cost effective way to explore the genome diversity of *P. malariae* from unprocessed blood of infected individuals. Further studies should consider the analysis of a larger number of samples from a greater geographical range and different clinical outcomes, in addition to studies investigating the subtelomeric regions with long read technologies. Such studies are necessary to characterize the epidemiology and genetic diversity of *P. malariae* populations, with the potential to provide biological insights for disease control.

## Methods

### Ethics statement

Isolated from Thailand were collected with ethical approval from the Mahidol Faculty of Tropical Medicine Ethics Committee (Ref: 2015-001.01); PHE-MRL samples are analysed under NHS Ethics approval (#18/LO/0738). In both instances, samples were collected according to relevant guidelines and regulations in both Thailand and the UK, and informed consent was obtained for all subjects over the age of 18 (for subjects under 18 years old, consent was obtained from the appropriate legal guardian.

### Sample collection and processing

This project used nine *P. malariae* DNA samples extracted from unprocessed venous blood from infected individuals in Thailand. Parasite density (parasites/µl) determined by microscopy was available for these isolates. Genomic DNA was extracted from frozen unprocessed blood using the QIAamp DNA Blood Mini Kit (Qiagen) or the QIAsymphony DSP DNA Kit in combination with a QIAsymphony SP instrument (Qiagen), according to manufacturer’s instructions. As microscopy is prone to human errors, all extracted DNA samples were subject to qPCR as outlined by Shokoples et al*.*^36^ to ensure that only *P. malariae* single species infections were used.

A further ten DNA samples were provided by the Public Health England-Malaria Reference Laboratory (PHE-MRL) at the London School of Hygiene and Tropical Medicine (LSHTM). These samples were sourced from individuals who had reported recent travel to only one country with malaria transmission, including: Kenya (n = 2), Liberia (n = 2), Sierra Leone (n = 2), Sudan (n = 1) and Uganda (n = 3) between 2010 and 2017. PHE-MRL samples are commonly sourced from individuals returning to visit relatives in their original native country. For species identification, PHE-MRL samples perform both a nested PCR^52^ and qPCR^36^ and are archived according to the species present.

Total DNA concentration for all samples was quantified using a Qubit v2.0 fluorometer (Thermo Fisher Scientific).

### Selective whole genome amplification

The *swga* program (www.github.com/eclarke/swga) was used to identify primers that preferentially amplify the *P. malariae* genome^35^, using its reference genome (PmUG01, https://plasmodb.org) as the target (foreground), and the human genome (GRCh37; https://grch37.ensembl.org/) as the background. The *swga* program ranks primers dependant on the ratio of foreground genome binding to the background genome binding, combined with the evenness of primer binding along the target genome and generates multiple potential primer sets. The five highest-ranked sets consist of combinations of 4 to 6 oligonucleotides each, with overlapping primers. The set that ranked highest (Pmset1) consisted of five primers: TATGTATA*T*T, TTATTC*G*T, TTCGTT*A*T, TTTTTA*C*G, TATTTC*G*T, that were ordered with a phosphorothioate bond (represented by *) modifications to prevent primer degradation by the exonuclease activity of the Phi29 polymerase. To evaluate the efficacy of Pmset1 for SWGA of the *P. malariae* genome, we tested two samples (PM_THA_001 and PM_THA_002) and sequenced both before and after SWGA.

DNA samples were subject to SWGA following previously published protocols^31,32,37^. All SWGA reactions were carried out in a UV Cabinet for PCR Operations (UV-B-AR, Grant-Bio) to eliminate potential contamination. Briefly, a maximum of 60 ng of gDNA (minimum of 5 ng) was added to a total 50 µl reaction alongside 5 µl of 10 × Phi29 DNA Polymerase Reaction Buffer (New England BioLabs), 0.5 µl of Purified 100 × BSA (New England BioLabs), 0.5 µl of 250 µM Primer mix, 5 µl 10 mM dNTP (Roche), 30 units Phi29 DNA Polymerase (New England BioLabs) and Nuclease-Free Water (Ambion, The RNA Company) to reach a final reaction volume of 50 µl. The reaction was carried out on a thermocycler with the following step-down program: 5 min at 35 °C, 10 min at 34 °C, 15 min at 33 °C, 20 min at 32 °C, 25 min 31 °C, 16 h at 30 °C and 10 min at 65 °C. After successful validation of Pmset1, the remaining samples underwent SWGA as described above. After SWGA, samples were purified using a 1:1 ratio of AMPure XP beads (Beckman-Coulter), following manufacturer’s instructions.

### Library preparation and WGS

SWGA samples and the unamplified negative controls were sequenced on either an Illumina MiSeq or HiSeq4000 platform. For the MiSeq runs, the QIAseq FX DNA Library Kit (QIAGEN) was used for library preparation according to the manufacturer’s protocol, with a 20-min fragmentation step. For the HiSeq4000 runs, samples were prepared using the NEB Next Ultra DNA Library Prep Kit for Illumina (from New England BioLabs Inc., E7370). Library DNA concentration was analysed using a Qubit 2.0 fluorometer. All sequencing reactions were performed using paired (2×) 150 bp reads.

### Sequence data analysis

Raw fastq files were trimmed using trimmomatic set to default parameters^53^, and aligned to the *P. malariae* UG01 reference genome (PlasmoDB) using *bwa-mem* software^54^. SNPs were identified using the *samtools* software suite (samtools.sourceforge.net)^55^ and filtered for quality based on previously described methods^56^. The coverage of each nucleotide position was analysed using *sambamba*^57^, which was set to include only SNPs with coverage levels of at least fivefold. Poor quality samples were removed (< 40% of the genome covered by 5 reads) to leave 18 high quality samples. We used estMOI^38^ to determine MOI for samples, and the major allele was used when heterozygous SNP calls were found.

### Determining and excluding subtelomeric regions

To exclude hypervariable subtelomeric regions the *P. malariae* genome was split into 5 kb segments and the average number of SNPs was calculated. We defined an upper limit for the number of SNPs within each window in order to identify highly polymorphic windows. This SNP limit was used in conjunction with the positions of the *Pm-fam* gene families to define the subtelomeric regions of each chromosome and exclude these from downstream analysis.

### Population genetics

To investigate the population structure of *P. malariae* parasites, a distance matrix was created which was based on a matrix of pairwise identity calculated from the SNPs present in each sample. Using the distance matrix, a maximum likelihood tree was produced using *Iqtree*^39^ with *Modelfinder*^40^ to select the best model of substitution and ultrafast bootstrap analysis^41^. The resulting Newick tree was visualised in iTOL^42^. The nucleotide diversity (π) metric was used to investigate the genetic variability between samples, and was calculated using the *pegas* (v0.10) package^58^, which defines nucleotide diversity as the average number of SNPs per position between two sequences.

### Drug resistance orthologs

Orthologs of known genes involved in drug resistance in *P. falciparum* were analysed. The SNPs were described using the *snpEff* software^59^ which annotates the genes affected, the type of mutation, and if non-synonymous, the amino-acid change that has occurred. The coverage of genes of interest was also analysed using the output file from applying *sambamba* software^57^. The genes investigated and their respective IDs are summarised in S5 Table.

## Supplementary information

Supplementary information

## Data Availability

All raw sequence data is listed in the European Nucleotide Archive (study accession number PRJEB33837).
