# Supplementary material for: Selective whole genome amplification of Plasmodium malariae DNA from clinical samples reveals insights into population structure
Source: Sci Rep. 2020 Jul 2;10:10832. doi: 10.1038/s41598-020-67568-4 (PMC7331648; doi:10.1038/s41598-020-67568-4)
Supplement: Supplementary file 1 — Supplementary information [file 41598_2020_67568_MOESM1_ESM.pdf]

Selective Whole Genome Amplification of *Plasmodium malariae* DNA from clinical samples reveals insights into population structure

Amy Ibrahim[1], Ernest Diez Benavente[1], Debbie Nolder[1,2], Stephane Proux[3], Matthew Higgins[1], Julian Muwanguzi[1,2], Paula Josefina Gomez Gonzalez[1], Hans-Peter Fuehrer[4], Cally Roper[1], Francois Nosten[3,5], Colin Sutherland[1], Taane G. Clark[1,6], Susana Campino[1]

SUPPLEMENTARY INFORMATION

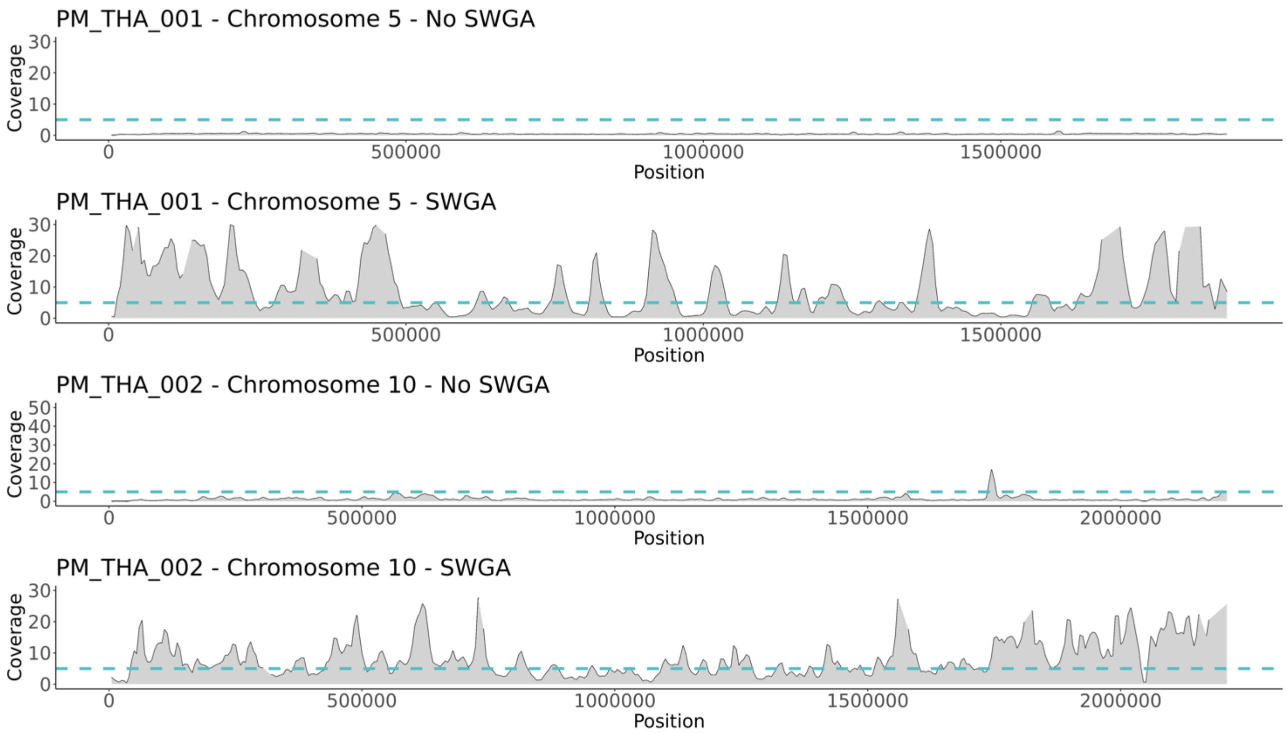

S1 Fig. Genome coverage is increased after SWGA. The top two plots show the coverage (as measured by the number of sequencing reads at each position) of chromosome 5 for sample PM_THA_001 before (No SWGA) and after amplification with Pmset1 (SWGA). The same comparison is shown below with chromosome 10 for sample PM_THA_002. Coverage was calculated using a sliding window of 10 kb. The horizontal y-intercept indicates a coverage of 5-fold which is recommended for confident SNP calling.

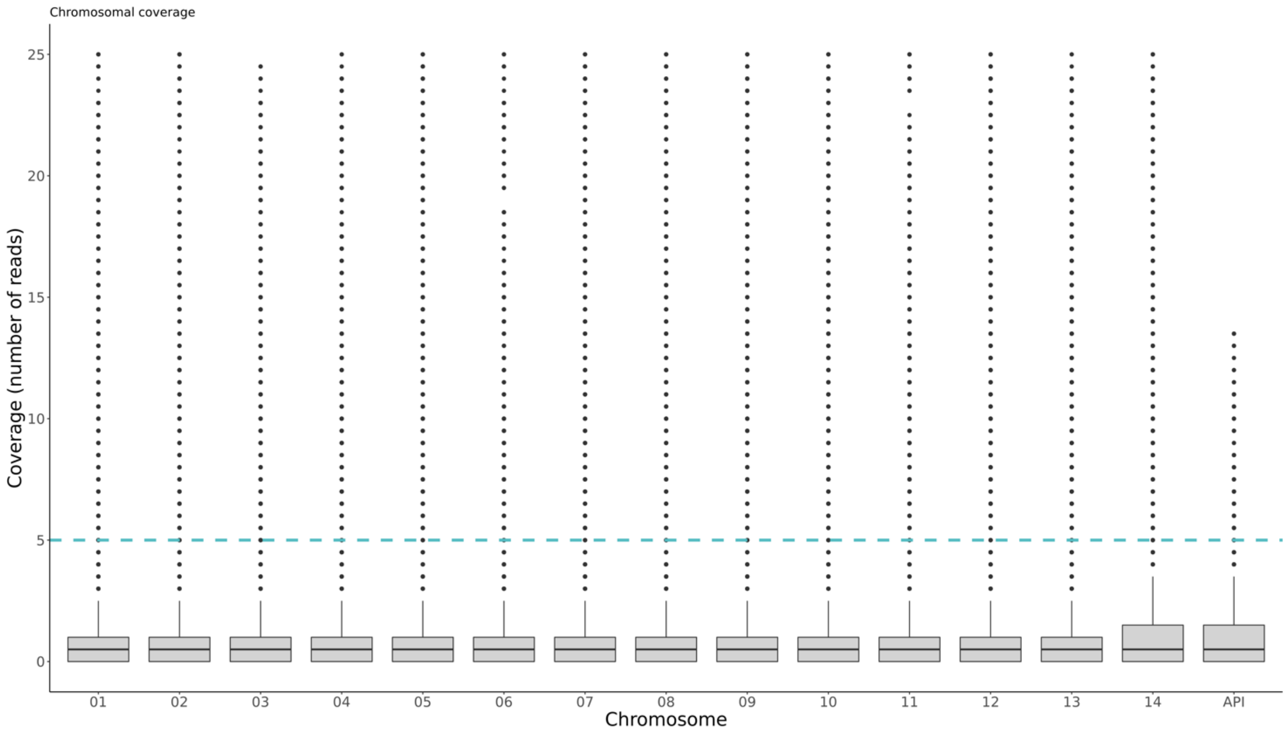

S2 Fig. Sequencing coverage by chromosome without amplification. The distribution of coverage for each position within the genome between two unamplified samples (PM_THA_001 and PM_THA_002), among the 14 nuclear chromosomes and the organellar apicoplast genome. The blue horizontal dashed line represents a cut-off of coverage of 5-fold which is recommended for SNP calling. Outliers are denoted by a black circle, many individual positions will have a coverage of 5-fold, but the majority of the genome falls below this.

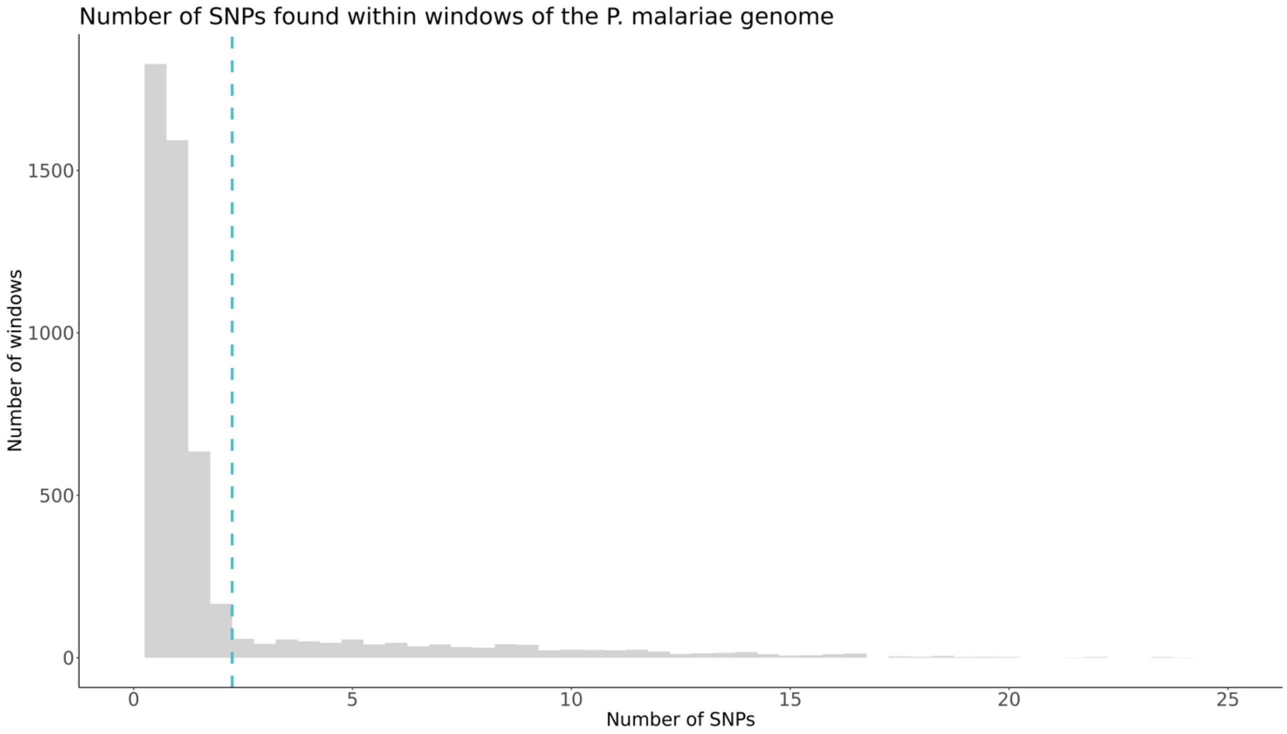

S3 Fig. Number of SNPs seen within 5 kb windows of the *P. malariae* genome. An average of the number of SNPs seen within all 18 samples was generated for 5 kb sliding windows across the entire genome. The distribution of SNPs within each window is shown. The blue dashed x-intercept is at 2.25 SNPs which is used as the limit of SNPs per window for the core genome.

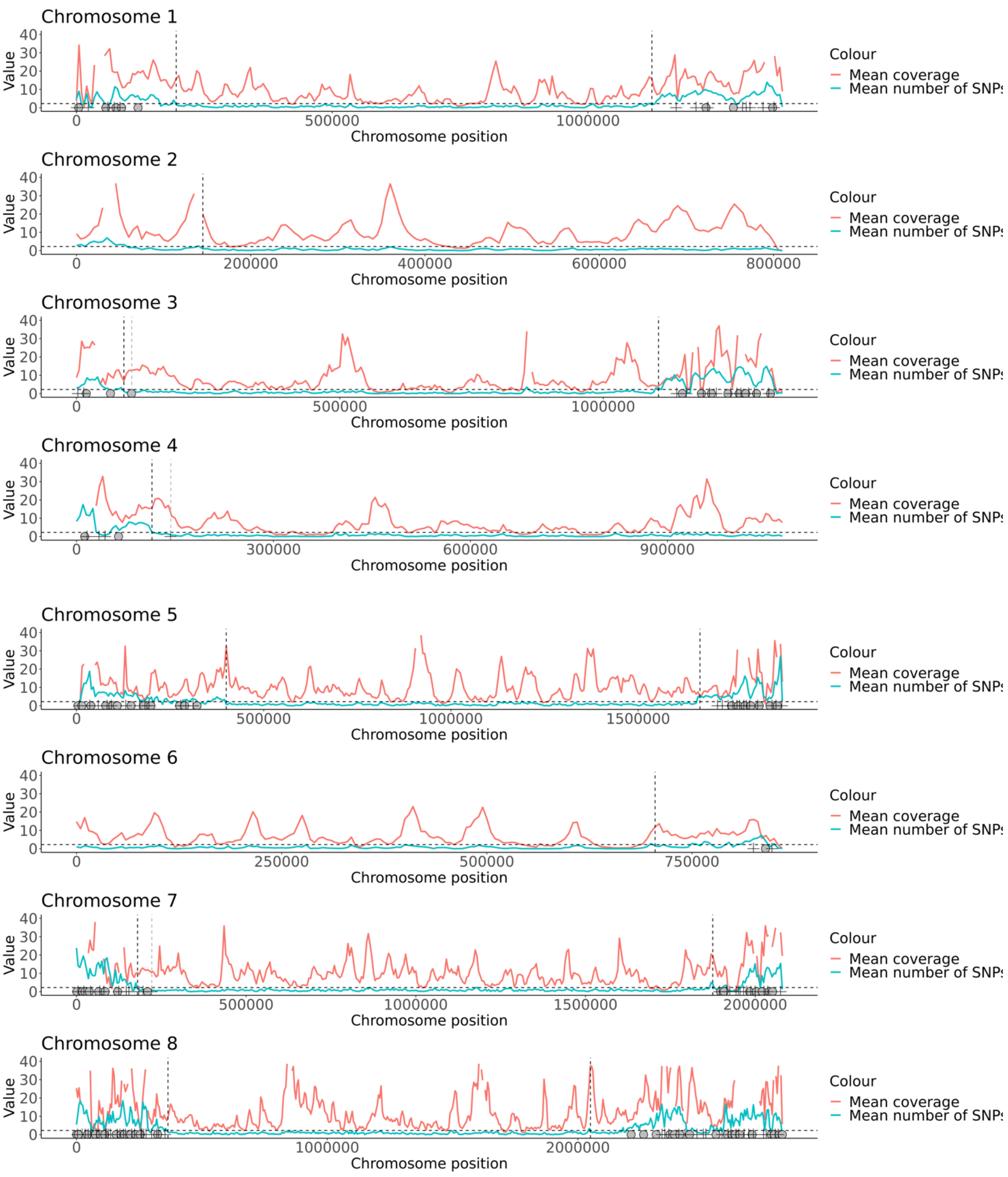
Chromosome 1
Value
40 30 20 10 0
0 500000 1000000
Chromosome position
Colour
Mean coverage
Mean number of SNP
Chromosome 2
Value
40 30 20 10 0
0 200000 400000 600000 800000
Chromosome position
Colour
Mean coverage
Mean number of SNP
Chromosome 3
Value
40 30 20 10 0
0 500000 1000000
Chromosome position
Colour
Mean coverage
Mean number of SNP
Chromosome 4
Value
40 30 20 10 0
0 300000 600000 900000
Chromosome position
Colour
Mean coverage
Mean number of SNP
Chromosome 5
Value
40 30 20 10 0
0 500000 1000000 1500000
Chromosome position
Colour
Mean coverage
Mean number of SNP
Chromosome 6
Value
40 30 20 10 0
0 250000 500000 750000
Chromosome position
Colour
Mean coverage
Mean number of SNP
Chromosome 7
Value
40 30 20 10 0
0 500000 1000000 1500000 2000000
Chromosome position
Colour
Mean coverage
Mean number of SNP
Chromosome 8
Value
40 30 20 10 0
0 1000000 2000000
Chromosome position
Colour
Mean coverage
Mean number of SNP

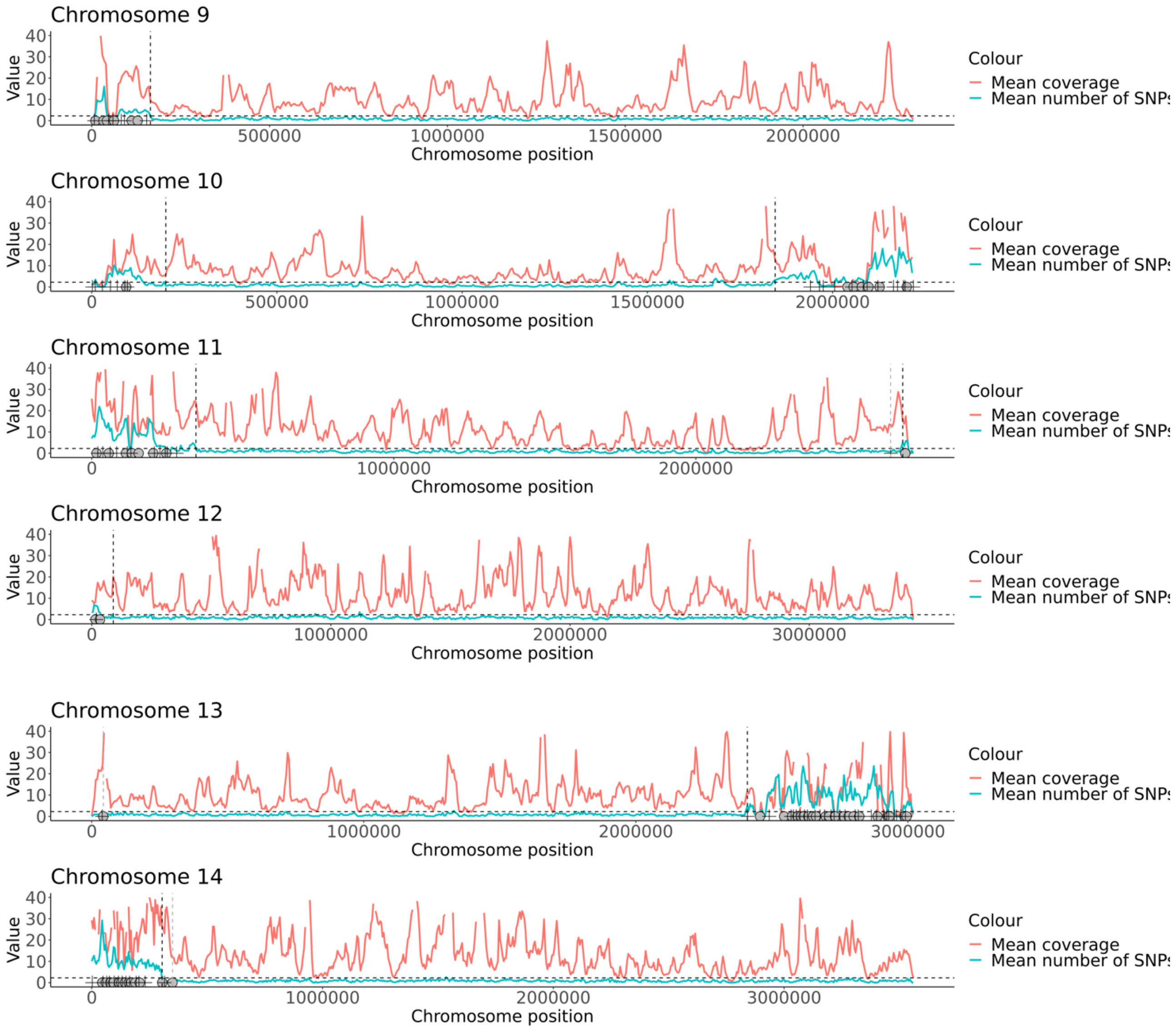

S4 Fig. Determining and excluding hypervariable subtelomeric regions. The average coverage (pink) and number of SNPs (blue) between all 18 samples for each nuclear chromosome. The black dashed horizontal line demonstrates the previously chosen SNP limit per 5 kb window (as defined in **S3 Fig.**). Black dashed lines are placed at the suggested hypervariable region cut-off points, where clusters of windows demonstrating >2.25 SNPs are seen. The midpoints of *Pm-fam* gene families are annotated; *Pm-fam-l* gene positions are denoted by a black plus, whilst *Pm-fam-m* gene positions are denoted with a grey circle. There are cases where *Pm-fam* gene families are located outside of the hypothesised hypervariable regions using the 2.25 SNP limit per window, and in these instances, the terminal or initial position of the *Pm-fam* gene is located with a grey dashed line and this is used as the amended hypervariable cut-off position (see **S3 Table** for coordinates).

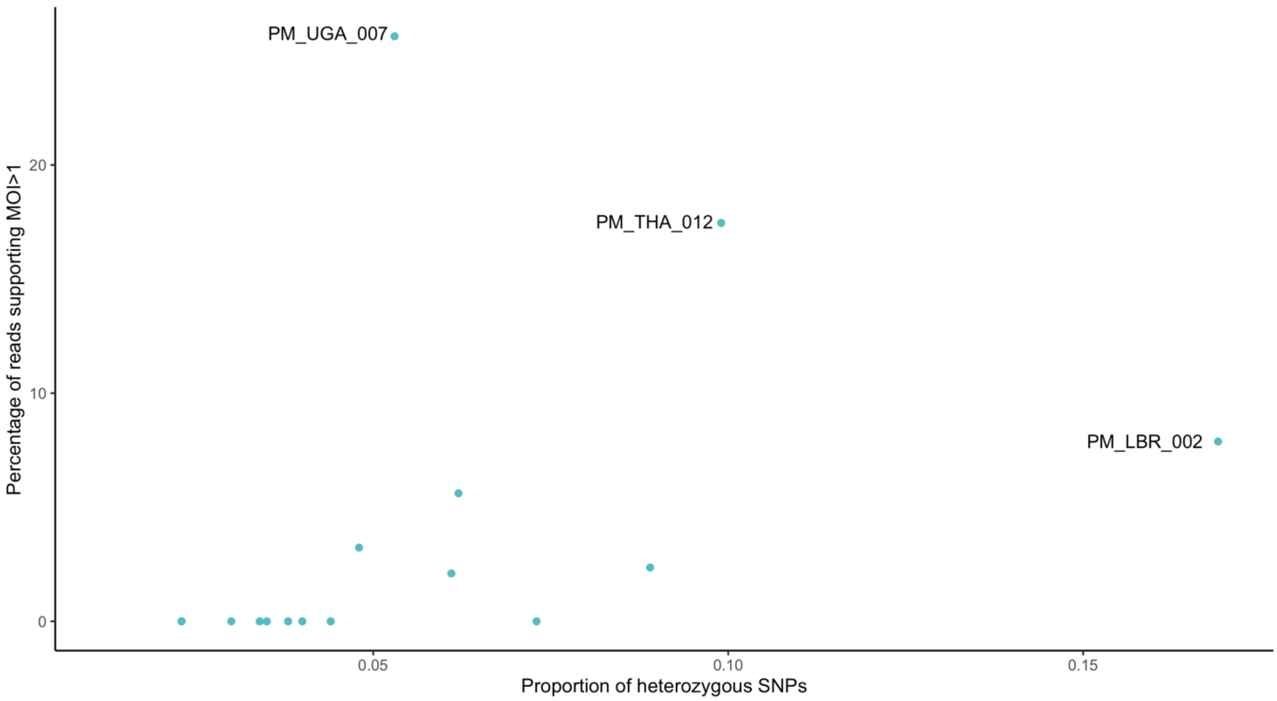

S5 Fig. Determining multiplicity of infection (MOI). EstMoi was ran for all sequenced samples, values were obtained for 18 samples (not including PM_KEN_001, PM_LBR_003 and PM_UGA_001 which did not have sufficient SNP density for estMOI) (**S2 Table**). Three samples appear to separate from the main cluster of samples (PM_LBR_002, PM_THA_012 and PM_UGA_007).

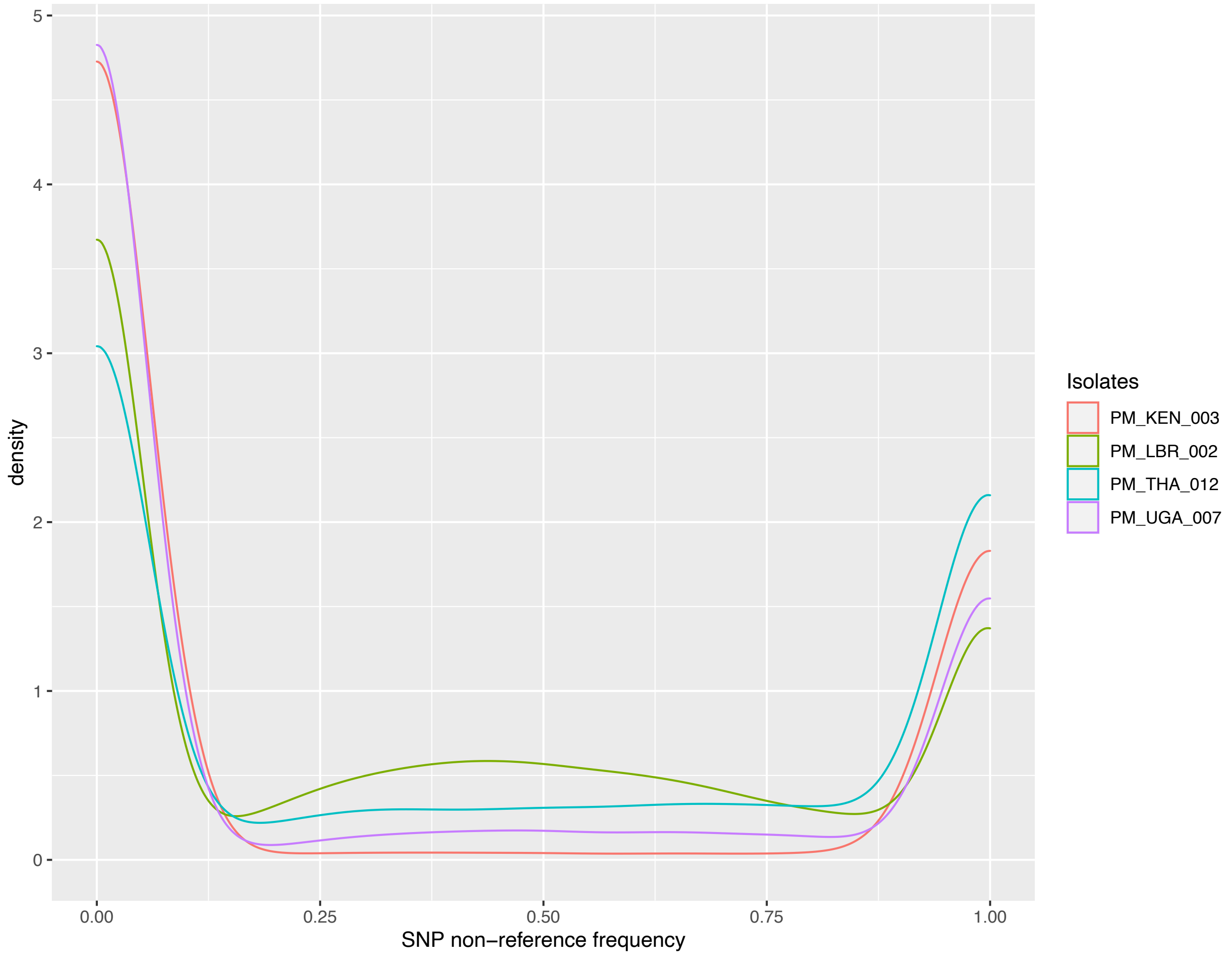

S6 Fig. Density plot of non-reference MAF distribution for isolates with MOI>1*.*

Distribution (density plot) of non-reference minor allele reference (MAF) for isolates with MOI>1 according to estMOI (PM_LBR_002, PM_THA_012, PM_UGA_007). Isolate PM_KEN_003 did not present evidence of MOI>1 and has been included as reference. The density (y-axis) is scaled so that the area under the curve adds up to 1.

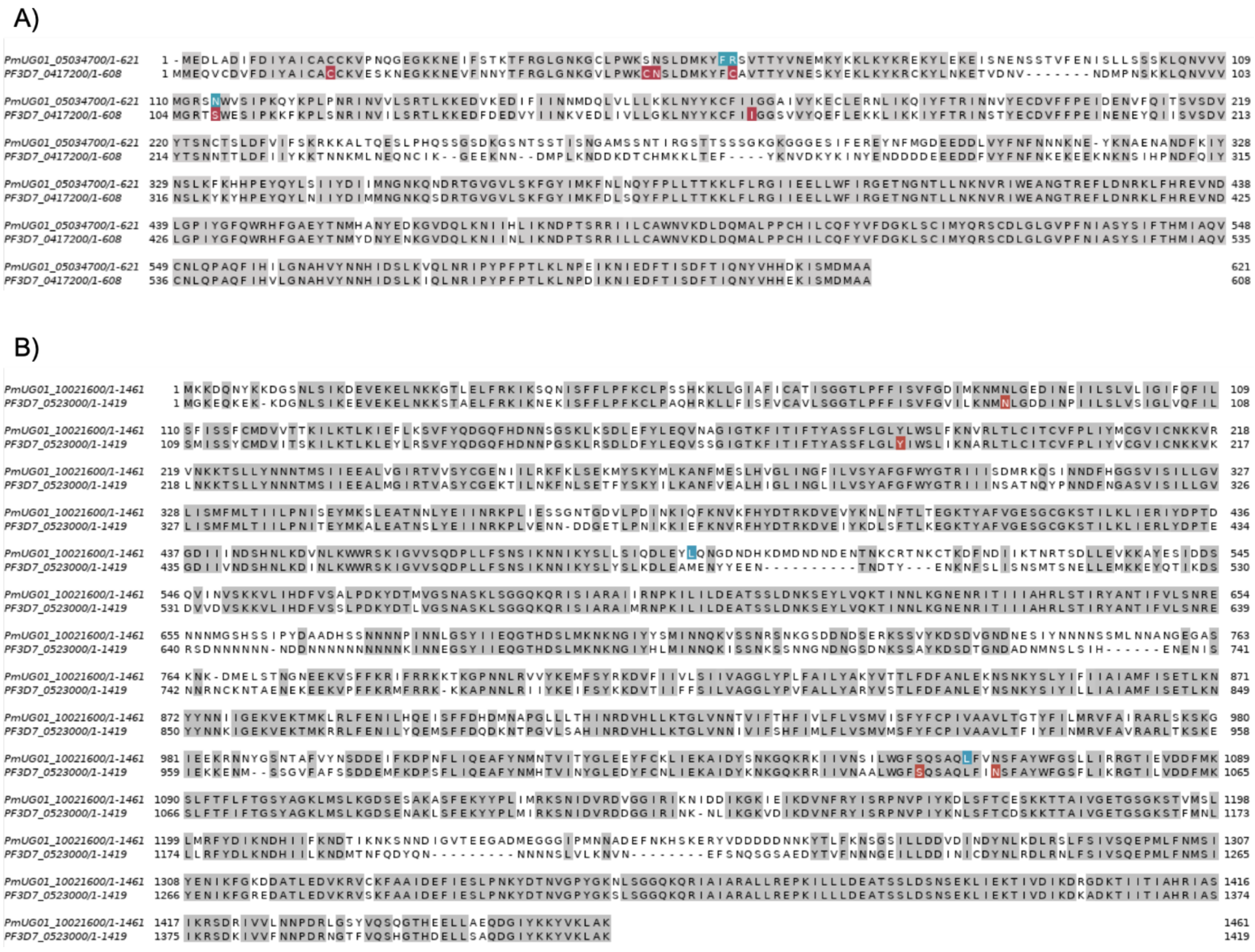

S7 Fig. Substitutions within *P. malariae* orthologs of drug resistance-associated loci in *P. falciparum*.

Amino acid sequences aligned using Clustal Omega. Regions that are conserved between the two positions are highlighted in grey. Positions associated with drug resistance mutations within *P. falciparum* are highlighted in red. Predicted amino acid substitutions found in our *P. malariae* sample set are highlighted in blue. **A)** *PMDHFR* and *PFDHFR.* Both positions 57-58 and 114 within *PMDHFR* lie close to amino acid alterations associated with drug resistance in *P. falciparum (*C59R and S108N/T)*.* **B)** *PMMDR1* and *PFMDR1*. The amino acid substitution at position 1,063 within PmUG01_10021600.1 lies in close proximity to the N1042D substitution associated with quinine resistance.

S1 Table. Pmset1 primers. The sequences primers within Pmset1 are demonstrated. The asterisk in the primer sequence denotes the position of a phosphorothioate bond which inhibits primer degradation. Binding sites within the human genome were calculated using the human GRCh37 genome sequence available at http://grch37.ensembl.org and binding sites within the *P. malariae* genome were calculated using the PmUG01 reference genome available at https://plasmodb.org.

Primer set	Primer sequence	Binding sites in human genome	Binding sites in *P. malariae* genome
Pmset1	TATGTATA*T*T	6054	3057
	TTATTC*G*T	3475	3040
	TTCGTT*A*T	4381	3088
	TTTTTA*C*G	3991	3863
	TATTTC*G*T	3961	3863
	Total Binding Sites	***21862***	***16521***

S2 Table. Sequencing statistics for all samples. Sequence analysis data for all samples. Samples highlighted in grey are comparative samples before SWGA, this is also indicated in the 'Amplification' column. Samples that were excluded due to poor quality and not used in downstream population genetics analysis are labelled with an (E). Accession codes are given for each sample and can be found using the project code (PRJEB37746). Multiplicity of infection (MOI) was calculated using estMOI and the percentage of reads supporting a MOI>1 are given.

Sample	Accession code	Country	Amplified DNA	Pm CT-value (qPCR data)	Parasites/ul (% Parasitaemia)	Total reads	Mapped reads	% Mapped reads	% Genome coverage ≥5	% Genome coverage ≥10	Mean coverage	Mean genic coverage	Median genic coverage	Mean intergenic coverage	Median intergenic coverage	Mean mitochondria coverage	Unfiltered SNPs	Core SNPs filtered	% Genome with MOI>1
PM_THA_001 (E)	/	Thailand	No_SWGA	31.74	256 (0.0064)	6264676	1005761	16.05	0.86	0.12	0.45	0.44	0	0.47	0	/	3	/	/
PM_THA_001	ERS4423439	Thailand	SWGA	31.74	256 (0.0064)	7174584	3154695	43.97	44.17	25.28	8.57	6	3	11	4	23.78	41962	3256	0
PM_THA_002 (E)	/	Thailand	No_SWGA	27.73	576 (0.0144)	5073136	419922	8.28	5.01	0.45	1.25	1.66	1	0.89	0	/	49	/	/
PM_THA_002	ERS4423440	Thailand	SWGA	27.73	576 (0.0144)	8036914	4250708	52.89	64.06	36.16	12.59	16	7	10	6	338.52	43237	6089	0
PM_THA_003	ERS4423441	Thailand	SWGA	30.59	8096 (0.2024)	7852281	4215247	53.68	63.46	40.97	14.63	13	6	16	8	3789.74	45869	6567	2.10
PM_THA_005	ERS4423442	Thailand	SWGA	23.60	6464 (0.1616)	8028153	5735625	71.44	88.31	72.65	22.68	23	18	22	15	1533.33	57182	9998	2.36
PM_THA_006	ERS4423443	Thailand	SWGA	23.72	5312 (0.1328)	5584655	5130545	91.87	75.09	53.23	19.13	14	9	24	12	238.56	56707	6826	0
PM_THA_009a* (E)	ERS4423444	Thailand	SWGA	31.20	64 (0.0016)	3684878	2765795	75.06	44.05	25.70	9.28	7	3	11	4	/	43853	/	0
PM_THA_009b* (E)	ERS4423445	Thailand	SWGA	31.20	64 (0.0016)	2868636	2327144	81.12	27.32	15.84	6.41	4	1	8	2	/	37417	/	0
PM_THA_009_com	ERS4423446	Thailand	SWGA	31.20	64 (0.0016)	6553515	5092953	77.71	52.44	34.66	15.69	11	4	20	6	294.51	48212	4698	0
PM_THA_010	ERS4423447	Thailand	SWGA	23.28	1648 (0.0412)	6934812	4135980	59.64	83.89	62.05	16.49	16	13	17	12	884.78	43804	9042	0

PM_THA_011	ERS4423448	Thailand	SWGA	25.20	16 (0.0004)	6179776	2257408	36.53	63.10	31.03	8.47	8	7	9	6	164.40	43804	5629	0
PM_THA_012	ERS4423449	Thailand	SWGA	24.42	544 (0.0136)	6909366	3264421	47.25	78.03	50.69	12.71	13	10	13	9	414.93	52348	8147	17.46
PM_KEN_001	ERS4423432	Kenya	SWGA	/	/	5709031	3454616	60.51	72.05	44.58	12.03	11	8	13	9	59.60	50899	4820	/
PM_KEN_003	ERS4423433	Kenya	SWGA	/	/	6744405	2186998	32.43	53.11	24.02	7.44	7	5	8	5	28.76	44678	3400	0
PM_LBR_002	ERS4423434	Liberia	SWGA	/	/	7180349	4242520	59.09	86.46	66.06	17.10	17	14	17	13	309.94	63484	8474	7.88
PM_LBR_003 (E)	ERS4423435	Liberia	SWGA	/	/	9338062	5627043	60.26	0.07	0.03	0.19	0	0	0	0	/	25	/	/
PM_SDN_001	ERS4423436	Sudan	SWGA	/	/	5369713	4461837	83.09	72.52	50.32	16.96	13	8	20	11	419.30	54943	5154	0
PM_SLE_002	ERS4423437	Sierra_Leone	SWGA	/	/	8039293	4286570	53.32	84.15	62.52	16.46	17	13	16	12	258.93	53739	7034	5.61
PM_SLE_004	ERS4423438	Sierra_Leone	SWGA	/	/	6933075	1513093	21.82	44.96	14.67	5.35	6	4	5	4	184.48	28204	2554	0
PM_UGA_001	ERS4423451	Uganda	SWGA	/	/	2846843	1666685	58.55	46.56	17.59	5.90	5	4	6	4	50.56	37419	2534	/
PM_UGA_003	ERS4423452	Uganda	SWGA	/	/	6500133	3316826	51.03	81.84	53.76	13.22	13	11	13	10	373.45	53571	6525	3.23
PM_UGA_007	ERS4423453	Uganda	SWGA	/	/	6673599	2481168	37.18	58.43	32.44	9.48	9	6	10	6	87.20	48414	3836	25.64

S3 Table. Identification of core nuclear genome positions. Not all chromosomes have annotated *Pm-fam* genes or hypervariable regions at both ends of the chromosomes, these chromosomes are indicated with an asterisk.

Chromosome	Start	End	Classification
PmUG01_01_v1	0	194999	Hypervariable
PmUG01_01_v1	195000	1125000	Core
PmUG01_01_v1	1125001	1381517	Hypervariable
PmUG01_02_v1*	0	59999	Hypervariable
PmUG01_02_v1*	60000	813519	Core
PmUG01_03_v1	0	89999	Hypervariable
PmUG01_03_v1	90000	1105000	Core
PmUG01_03_v1	1105001	1341933	Hypervariable
PmUG01_04_v1*	0	143680	Hypervariable
PmUG01_04_v1*	143681	1076824	Core
PmUG01_05_v1	0	399999	Hypervariable
PmUG01_05_v1	400000	1665000	Core
PmUG01_05_v1	1665001	1887011	Hypervariable
PmUG01_06_v1*	0	705000	Core
PmUG01_06_v1*	705001	862289	Hypervariable
PmUG01_07_v1	0	221927	Hypervariable
PmUG01_07_v1	221928	1875000	Core
PmUG01_07_v1	1875001	2080590	Hypervariable
PmUG01_08_v1	0	364999	Hypervariable
PmUG01_08_v1	365000	2050000	Core
PmUG01_08_v1	2050000	2818517	Hypervariable
PmUG01_09_v1*	0	164999	Hypervariable
PmUG01_09_v1*	165000	2312276	Core
PmUG01_10_v1	0	199999	Hypervariable
PmUG01_10_v1	200000	1854000	Core
PmUG01_10_v1	1854001	2219074	Hypervariable
PmUG01_11_v1	0	344999	Hypervariable
PmUG01_11_v1	345000	2644881	Core
PmUG01_11_v1	2644882	2721161	Hypervariable
PmUG01_12_v1*	0	89999	Hypervariable
PmUG01_12_v1*	90000	3436769	Core
PmUG01_13_v1	0	42699	Hypervariable
PmUG01_13_v1	42700	2410000	Core
PmUG01_13_v1	2410001	3023685	Hypervariable
PmUG01_14_v1	0	350186	Hypervariable
PmUG01_14_v1	350187	3564280	Core

S4 Table. Pairwise genome-wide nucleotide diversity (π; x10^{-4}). Nucleotide diversity values are coloured to demonstrate samples with high or low diversity in comparison to other samples. Low diversity is shaded in yellow and pale orange, increasing to darker orange for high diversity (yellow for 0 to 1, pale orange for 1 to 2, medium orange for 1 to 2, and dark orange for 2 to 4; all x10^{-4}).

Region		Thailand									East Africa						West Africa		
	Samples	PM_THA_001	PM_THA_002	PM_THA_003	PM_THA_005	PM_THA_006	PM_THA_009com	PM_THA_010	PM_THA_011	PM_THA_012	PM_KEN_001	PM_KEN_003	PM_UGA_001	PM_UGA_003	PM_UGA_007	PM_SDN_001	PM_LBR_002	PM_SLE_002	PM_SLE_004
**Thailand**	PM_THA_001	/	2.1	2.23	2.29	2.04	1.75	1.99	1.64	1.87	2.75	2.79	2.35	2.91	2.64	2.72	3	3.05	2.41
	PM_THA_002	2.1	/	0.36	2.52	2.16	2.13	2.32	2.04	1.94	3.06	3.1	2.59	3.19	2.91	3.08	3.26	3.33	2.75
	PM_THA_003	2.23	0.36	/	2.52	2.25	2.18	2.34	2.1	1.98	3.12	3.12	2.67	3.21	2.96	3.12	3.25	3.3	2.74
	PM_THA_005	2.29	2.52	2.52	/	2.38	2.21	2.49	2.18	2.1	3.25	3.26	2.95	3.53	3.17	3.37	3.64	3.68	3.06
	PM_THA_006	2.04	2.16	2.25	2.38	/	1.99	2.38	2.17	2.17	2.91	2.98	2.55	3.05	2.85	2.9	3.26	3.27	2.68
	PM_THA_009com	1.75	2.13	2.18	2.21	1.99	/	2.13	1.4	2.07	2.8	2.88	2.43	2.98	2.82	2.83	3.11	3.14	2.62
	PM_THA_010	1.99	2.32	2.34	2.49	2.38	2.13	/	1.94	2.23	3.22	3.24	2.78	3.39	3.11	3.26	3.44	3.55	2.89
	PM_THA_011	1.64	2.04	2.1	2.18	2.17	1.4	1.94	/	2.03	2.91	2.92	2.55	3.07	2.78	2.89	3.08	3.26	2.57
	PM_THA_012	1.87	1.94	1.98	2.1	2.17	2.07	2.23	2.03	/	3.08	3.06	2.67	3.2	2.91	3.09	3.23	3.38	2.72
**East Africa**	PM_KEN_001	2.75	3.06	3.12	3.25	2.91	2.8	3.22	2.91	3.08	/	0.27	1.87	2.45	2.09	2.26	2.48	2.43	1.89
	PM_KEN_003	2.79	3.1	3.12	3.26	2.98	2.88	3.24	2.92	3.06	0.27	/	1.93	2.4	2.08	2.33	2.5	2.47	1.89
	PM_UGA_001	2.35	2.59	2.67	2.95	2.55	2.43	2.78	2.55	2.67	1.87	1.93	/	2.07	1.86	1.94	2.11	2.17	1.69
	PM_UGA_003	2.91	3.19	3.21	3.53	3.05	2.98	3.39	3.07	3.2	2.45	2.4	2.07	/	2.2	2.45	2.71	2.78	2.19
	PM_UGA_007	2.64	2.91	2.96	3.17	2.85	2.82	3.11	2.78	2.91	2.09	2.08	1.86	2.2	/	2.15	2.34	2.38	1.88
	PM_SDN_001	2.72	3.08	3.12	3.37	2.9	2.83	3.26	2.89	3.09	2.26	2.33	1.94	2.45	2.15	/	2.47	2.59	2.01
West Africa	PM_LBR_002	3	3.26	3.25	3.64	3.26	3.11	3.44	3.08	3.23	2.48	2.5	2.11	2.71	2.34	2.47	/	2.71	2.09
	PM_SLE_002	3.05	3.33	3.3	3.68	3.27	3.14	3.55	3.26	3.38	2.43	2.47	2.17	2.78	2.38	2.59	2.71	/	2.06
	PM_SLE_004	2.41	2.75	2.74	3.06	2.68	2.62	2.89	2.57	2.72	1.89	1.89	1.69	2.19	1.88	2.01	2.09	2.06	/

S5 Table. Drug resistance associated genes in *P. falciparum* and their known orthologs in *P. malariae.*

Gene	*P. falciparum (3D7)*		*P. malariae (UG01)*	
	**Gene ID**	**Paper code**	**Gene ID**	**Paper code**
crt	PF3D7_070900	*Pfcrt*	PmUG01_01020700	*Pmcrt*
dhfr-ts	PF3D7_0417200	*Pfdhfr*	PmUG01_05034700	*Pmdhfr*
pppk-dhps	PF3D7_0810800	*Pfdhps*	PmUG01_14045500	*Pmdhps*
mdr1	PF3D7_052300	*Pfmdr1*	PmUG01_10021600	*Pmmdr1*
kelch13	PF3D7_1343700	*Pfk13*	PmUG01_12021200	*Pmk13*

S6 Table. WGS data from mixed infections. Four samples with mixed infections (multiple species) were sequenced and mapped to their respective reference genomes. The proportion of each parasite present was analysed using qPCR and the resulting cycle thresholds (CTs) are given. The following reference genomes are used: *P. malariae* (PmUG01), *P. vivax* (PvP01, https://plasmodb.org/common/downloads/Current_Release/PvivaxP01/fasta/data/) *P. ovale curtisi* (PocGh01, https://plasmodb.org/common/downloads/Current_Release/PovalecurtisiGH01/fasta/data/), *P. ovale wallikeri* (PowCR01 https://www.ebi.ac.uk/ena/data/view/ERS1452913).

Sample	Mapped_to	Pf_CT	Po_CT	Pm_CRT	Pv_CRT	Parasites/ul (%Parasitemia)	Total_reads	Map_reads	%_Mapped_reads	%_Genome_cov5	%_Genome_cov10
PM_THA_004	PmUG01	/	/	23.48	31.02	1520 (0.038)	7644777	4619930	60.433	85.004	66.919
PM_THA_004	PvP01	/	/	23.48	31.02	1520 (0.038)	7644777	37351	0.489	0.015	0.010
PM_THA_008	PmUG01	/	29.33	31.12	29.26	192 (0.0048)	3679905	305249	3.993	0.072	0.023
PM_THA_008	PocGH01	/	29.33	31.12	29.26	192 (0.0048)	3679905	1540021	41.849	25.825	15.677
PM_THA_008	PowENA	/	29.33	31.12	29.26	192 (0.0048)	3679905	117162	3.184	0.169	0.100
PM_THA_008	PvP01	/	29.33	31.12	29.26	192 (0.0048)	3679905	242561	6.592	0.046	0.024
PM_THA_013	PmUG01	/	33.43	26.31	/	4560 (0.114)	7533858	3928080	52.139	83.985	61.240
PM_THA_013	PocGH01	/	33.43	26.31	/	4560 (0.114)	7533858	106192	1.410	0.035	0.014
PM_THA_013	PowENA	/	33.43	26.31	/	4560 (0.114)	7533858	48638	0.646	0.034	0.018
PM_THA_020	PmUG01	/	26.72	28.33	26.70	4880 (0.122)	6917577	236151	3.414	0.048	0.020
PM_THA_020	PocGH01	/	26.72	28.33	26.70	4880 (0.122)	6917577	2605168	37.660	23.871	20.314
PM_THA_020	PowENA	/	26.72	28.33	26.70	4880 (0.122)	6917577	895565	12.946	28.435	12.095
PM_THA_020	PvP01	/	26.72	28.33	26.70	4880 (0.122)	6917577	391155	5.655	0.046	0.038
